# A paradigm of fragile Earth in Priestley's bell jar

**DOI:** 10.1186/2046-7648-1-4

**Published:** 2012-09-04

**Authors:** Daniel Martin, Andrew Thompson, Iain Stewart, Edward Gilbert, Katrina Hope, Grace Kawai, Alistair Griffiths

**Affiliations:** 1 UCL Centre for Altitude, Space and Extreme Environment Medicine, Portex Unit, Institute of Child Health, 30 Guilford Street, London, WC1N 1EH, UK; 2 Division of Surgery and Interventional Science, University College London, 9th Floor, Royal Free Hospital, London, NW3 2QG, UK; 3 BBC Television, Zone 2.20, BBC Pacific Quay, Glasgow, G51 1DA, UK; 4 School of Geography, Earth & Environmental Sciences, Plymouth University, Plymouth, PL4 8AA, UK; 5 Centre of Human & Aerospace Physiological Sciences, School of Biomedical Sciences, King's College London, London, SE1 1UL, UK; 6 The Eden Project, Bodelva, Cornwall, PL24 2SG, UK

**Keywords:** Atmosphere, Carbon dioxide, Oxygen, Hypoxia, Photosynthesis, Plants

## Abstract

**Background:**

Photosynthesis maintains aerobic life on Earth, and Joseph Priestly first demonstrated this in his eighteenth-century bell jar experiments using mice and mint plants. In order to demonstrate the fragility of life on Earth, Priestley's experiment was recreated using a human subject placed within a modern-day bell jar.

**Methods:**

A single male subject was placed within a sealed, oxygen-depleted enclosure (12.4% oxygen), which contained 274 C_3_ and C_4_ plants for a total of 48 h. A combination of natural and artificial light was used to ensure continuous photosynthesis during the experiment. Atmospheric gas composition within the enclosure was recorded throughout the study, and physiological responses in the subject were monitored.

**Results:**

After 48 h, the oxygen concentration within the container had risen to 18.1%, and hypoxaemia in the subject was alleviated (arterial oxygen saturation rose from 86% at commencement of the experiment to 99% at its end). The concentration of carbon dioxide rose to a maximum of 0.66% during the experiment.

**Conclusions:**

This simple but unique experiment highlights the importance of plant life within the Earth's ecosystem by demonstrating our dependence upon it to restore and sustain an oxygen concentration that supports aerobic metabolism. Without the presence of plants within the sealed enclosure, the concentration of oxygen would have fallen, and carbon dioxide concentration would have risen to a point at which human life could no longer be supported.

## Background

The Earth supports a fragile ecosystem, and its inhabitants depend for their survival upon complex interactions between them, which have developed over billions of years. Imbalance of one component in this bionetwork can have far-reaching effects on organisms whose existence relies upon the presence of other species. Despite the ability to alter their environment in diverse ways, humans are reliant for their survival upon an element derived primarily from plants and produced by chlorophyll during photosynthesis, oxygen (O_2_).

Photosynthesis is arguably the single most important chemical process on our planet, and the first colour images captured of Earth from space revealed the vast green hues of the landmasses supporting plant life, confirming its dominance within our ecosystem. Using energy from sunlight, chlorophyll strips electrons from water molecules, which then convert atmospheric carbon dioxide (CO_2_) into carbon compounds, producing O_2_ as a byproduct. Whilst mechanisms that use alternative naturally available compounds to release energy exist, the abundance of water on the surface of the Earth meant that photosynthesis rapidly became the foremost bio-energetic pathway on the planet. During the early era of chlorophyll photosynthesis, approximately 2,400 million years ago [1], the atmosphere was rich in CO_2_, whilst O_2_ was scarce. As time progressed and photosynthetic species slowly overwhelmed the surface of the Earth, the concentration of O_2_ rose and eventually reached levels we are accustomed to today.

In the early 1770 s, Joseph Priestley conducted a series of experiments that led to the discovery of the intimate relationship between plant and animal life [2]. In his principal experiment, Priestley placed a mouse within a sealed jar and observed it to eventually perish. When repeated with sprigs of mint within the jar, neither did the animal die ‘nor was it at all inconvenient to a mouse’ [2]. He had made the breakthrough that plants produce a substance which is life-giving to animals and then went on to describe ‘dephlogisticated air’, which, thanks to the French chemist Antoine Lavoisier, soon became known as ‘oxygen’. The story of photosynthesis was completed in 1779 when a Dutchman, Jan Ingenhousz, demonstrated that the process by which plants produce O_2_ is dependent upon light.

We hypothesised that a human could survive within a sealed modern-day bell jar, even if the O_2_ concentration within was significantly reduced from the outset, provided that it contained sufficient plant matter to generate O_2_ and remove CO_2_ via photosynthesis.

## Methods

Formal ethical approval was not sought for this experiment as it was designed for the purpose of a television demonstration; consent was implied through the subject's involvement in the project and participation in the event. The Chair of the University College London Committee on the Ethics of Non-NHS Human Research approved this strategy. Prior to commencing the experiment, a full medical screening questionnaire was completed by the subject, and he was assessed by a physician with experience in high altitude and acute hypoxia research (DM). The protocol was explained to the subject in full, along with a description of the potential risks and safety measures in place. A standard resuscitation kit was available throughout the experiment, along with bottled supplemental oxygen. A physician trained in Advanced Life Support was also present outside the container throughout the experiment, with the ability to enter the container at any point should there be concerns regarding the welfare of the subject.

**Figure 1:**
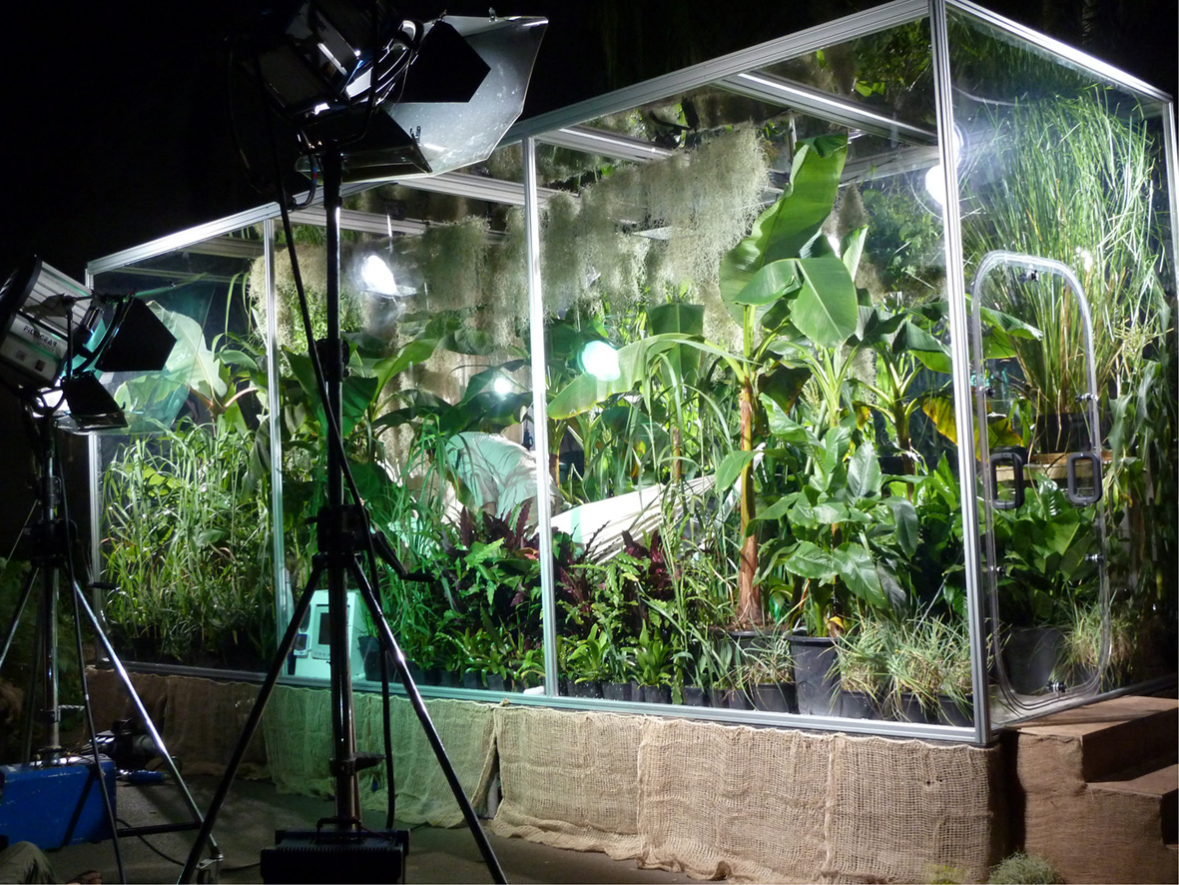
The sealed container with plants, the subject and external artificial lighting.

We constructed the first human recreation of Priestley's ‘mouse in a bell jar’ experiment to demonstrate the ability of plants to generate sufficient O_2_ to sustain human life in an enclosed environment [3]. A healthy 47-year-old male was placed within a transparent airtight container measuring 2.0 × 2.5 × 6.0 m (30 m^3^, Figure 1), itself placed within the rainforest biome at the Eden Project, Cornwall, UK. A selection of plants known for their high photosynthetic yield (under certain environmental conditions) was placed within the container. Prior to the experiment, containerised plants were grown in a peat-free Eden Project Melcourt mix within a standard glasshouse at a relative humidity of 70% to 80% and temperature range of 15°C to 30°C. During this growing phase, the plants were watered with liquid nutrient feed at 20 ml/L (N 177 ppm, P 35 ppm, K 119 ppm, Ca 49 ppm, Mg 17 ppm, B 0.2 ppm, Cu 0.08 ppm, Fe 1.44 ppm, Mn 0.48 ppm, Mo 0.04 ppm and Zn 0.64 ppm). In total, 274 plants consisting of 18 different taxa were placed within the container, with 10,967 leaves (excluding *Tillandsia usneoides*) and a total leaf area of 1,106,033 cm^2^ (Table 1). A mixture of C_3_ (ribulose diphosphate carboxylase utilising) and C_4_ (phosphoenolpyruvate carboxylase utilising) plants were selected in order to maximise photosynthetic potential within the container. Several C_4_ carbon fixation plants were grown, including *Miscanthus* x *giganteus* and *Zea mays* (maize), because they have advantages over C_3_ plants, resulting in superior carbon-gaining capacities and photosynthetic efficiency [4]. During the experiment, the subject regularly irrigated the plants when deemed necessary from a water source within the container.

**Table 1:** Taxa, number of leaves and leaf area of the plants placed within the container

**Scientific plant name**	**Common name**	**Number of plants**	**Total number of leaves**	**Total upper leaf area (cm**^**2**^**)**^**a**^
*Z. mays*	Corn or maize	25	375	37,192
*M.* x *giganteus*	Miscanthus	30	720	72,841
*Leymus arenarius*	Lyme grass	21	3,528	7,281
*Calathea rufibarba* ‘Wavestar’	Calathea	12	1,560	126,204
*Ctenanthe* ‘Golden Mosaic’	Ctenanthe	1	160	35,200
*Spathiphyllum* Cv.	Peace lily	3	282	214,656
*Saccharum officinarum*	Sugarcane	2	72	34,534
*Musa acuminata*	Banana	9	224	204,056
*Astelia chathamica* ‘Silver Spear’	Astelia	33	1,188	26,479
*Furcraea longaeva*	Fucraea	40	400	12,301
*Anthurium andreanum* Cv.	Flamingo flower	2	24	163,296
*Chrysopogon zizanioides*	Vetiver	2	1,088	116,873
*Aechmea* Cv.	Bromeliad	14	280	20,475
*Eruca sativa*	Rocket	16	272	11,288
*Lactuca sativa*	Lettuce	16	272	11,288
*Mentha spicata*	Mint	5	450	1,512
Ferns	Ferns	9	72	10,557
Totals		274	10,967	1,106,033

Individual leaf areas were determined by tracing a leaf onto graph paper; the area of the petiole was not included within the calculations. From each individual plant, a subsample representing three small, three medium and three large leaves were harvested, and the mean of each of the three leaves was taken and used to provide a representative small, medium and large leaf area. The number of small, medium and large leaves in each individual plant was then counted, and the corresponding areas were used to estimate the total leaf surface area. ^a^Total leaf area was calculated as both the upper and lower sides of the leaves. *T. usneoides*, a moss, was also placed within the container, but it was not possible to calculate leaf area.

In order to more clearly demonstrate oxygen production and highlight the effectiveness of photosynthesis in preserving human life, the environment within the container was rendered hypoxic at the start of the experiment. Three hypoxic generators (Hypoxico Everest Summit II, Hypoxico Inc, New York, NY, USA) were used to reduce the concentration of O_2_ in the container. These devices consist of a molecular sieve system that uses zeolite to separate nitrogen from O_2_ in the air and consequently provides a nitrogen-rich gas mixture to purge the atmosphere within the container. Connected to the container, and in conjunction with a one-way pressure relief valve, the hypoxic generators reduced the concentration of O_2_ to 12.4% prior to commencing the experiment. Once the subject was sealed inside the container and safety procedures had been confirmed, the hypoxic generators were switched off and the one-way valves were closed. Artificial lighting (8 × 2,000 W systems; ARRI, Munich, Germany) was placed around the container externally and switched on at the beginning of the experiment. A split air-conditioning unit (Clima 16 HP Portable Air Conditioner, Toshiba, Tokyo, Japan) was used to maintain temperatures for optimal plant growth and comfort for the subject whilst ensuring a sealed atmosphere. The concentrations of O_2_ and CO_2_ within the container were monitored with a gas analyser (Aspida, Analox, London, UK) and plotted every hour along with temperature and humidity from a digital hygro-thermometer (Brannan, Cumbria, UK). The subject's heart rate and arterial O_2_ saturation (SpO_2_) were monitored continuously (Johnson and Johnson Dinamap MPS Monitor and Onyx 9500, Nonin, Plymouth, MN, USA); respiratory rate was recorded hourly by manual calculation.

## Results

**Figure 2:**
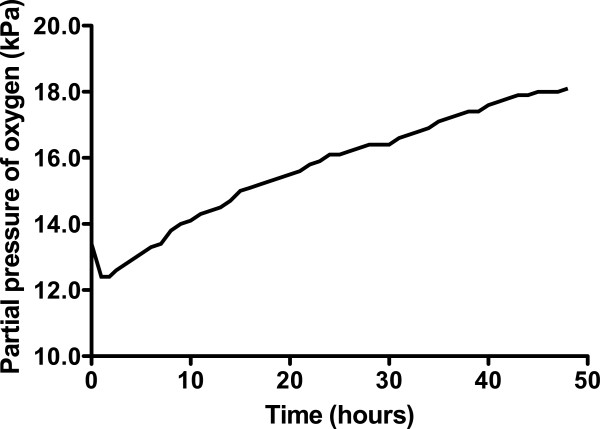
Change in oxygen concentration within the container over time.

**Figure 3:**
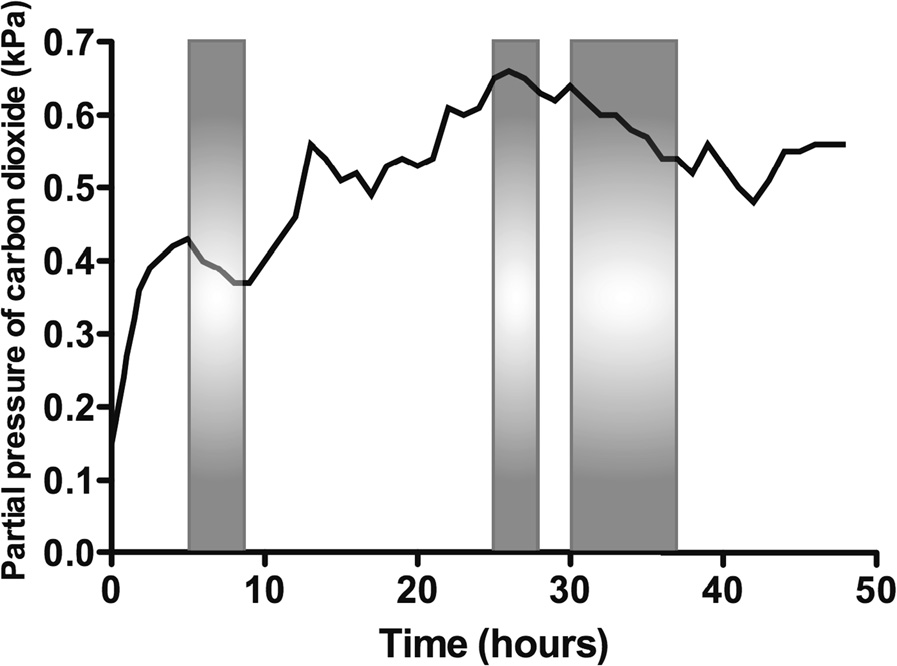
**Change in carbon dioxide concentration within the container over time.** The shaded areas are those during which the subject was sleeping.

**Figure 4:**
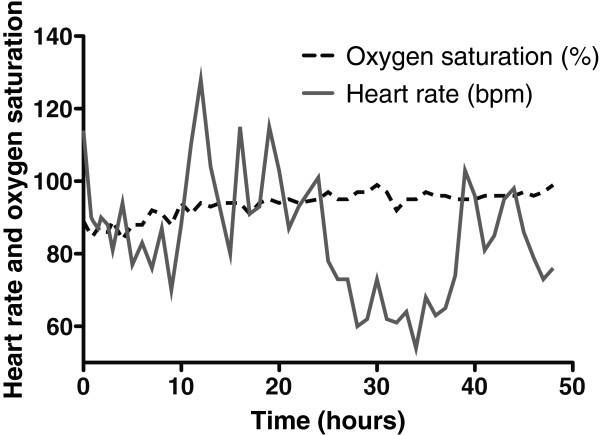
Changes in the subject's oxygen saturation and heart rate during enclosure within the container.

The concentration of O_2_ in the container rose throughout the experiment, peaking at 18.1% in the final hour (hour 48; Figure 2). The CO_2_ concentration fluctuated depending on the subject's activity within the container (declining noticeably during sleep), but there was an overall rise that peaked at 0.66%, approximately halfway through the experiment (Figure 3). There was a diurnal variation in temperature (25.3°C to 28.4°C), and humidity varied between 57% and 87%. On entering the hypoxic container, the subject had a heart rate of 90 beats per minute, respiratory rate of 20 breaths per minute and SpO_2_ of 86%. These figures returned to the subject's resting normal values as the concentration of O_2_ rose within the container. The subject's final SpO_2_ was 99% (Figure 4).

## Discussion

The design of the biological ecosystem in this study was such that human life was sustained for 48 h and the initial hypoxic environment restored to one of near-normal O_2_ concentration. In the early 1990's, the ‘Biosphere 2’ experiment was conducted to explore the feasibility of self-sustaining biospheres in space. This grand design consisted of a 200 m^3^ atmosphere within a dome that contained eight volunteers, which was designed to sustain them for 2 years [5]. However, the O_2_ concentration within the biosphere dropped from 20.9% to 14.2% after 16 months, so additional O_2_ had to be added to the atmosphere [6]. This decline was traced to a two-step process: firstly, there was O_2_ loss to organic soil matter producing CO_2_, and secondly, the CO_2_ was being captured by structural concrete to form calcium carbonate [5]. In the current experiment, the initial O_2_ concentration of 12.4% (equivalent to approximately 4,500 m above sea level) resulted in a marked reduction in the subject's SpO_2_ and represents an acute hypoxic exposure that is frequently associated with symptoms of altitude-related illness [7]. During the last few hours of the study, there was a small reduction in rate of the O_2_ concentration rise, perhaps due to deterioration in the condition of the plants, noticeable towards the end of the experiment. Direct heating and excessive light exposure, arguably both present in this experiment, can lead to the denaturing of enzymes within chlorophyll [8]. There were fluctuations in CO_2_ concentration throughout the study, with a tendency for it to rise as time progressed (Figure 3).

As well as providing an insight into the use of plants to maintain a self-sufficient biosphere, such as would be required on the surface of extra-terrestrial bodies without an atmosphere, our experiment highlights the detrimental effects of a markedly increased CO_2_ concentration. CO_2_ concentrations have altered dramatically over the course of the Earth's history [9], and there is much concern that levels are now rising at an alarming rate [10]. Under certain environmental conditions, increasing the ambient concentration of CO_2_ can be beneficial, increasing photosynthetic activity, plant growth and yield [11,12]. Using CO_2_ enrichment to increase plant growth and yield is now commonplace in commercial glasshouse crop production, with optimal levels being between 700 and 1,000 ppm [13]. However, in some species, super-elevated CO_2_ concentrations (over 2,000 ppm) induces foliar symptoms of chlorosis and necrosis [14,15], and levels above 10,000 ppm are known to cause damage to young maize plants after 48 h in the form of ‘yellow streaks’ [16]. During this experiment, the CO_2_ levels remained above 2,000 ppm and reached a maximum of 6,600 ppm, yet yellow streaks were observed on the maize plants by the end. It is possible that damage to the maize may have also reduced the photosynthetic yield and the production of O_2_ towards the end of this experiment. This study, therefore, provides an insight into the use of plants to maintain a self-sufficient biosphere, such as would be required on the surface of extra-terrestrial bodies without an atmosphere, and the potentially detrimental effects of a dramatically increased CO_2_ concentration.

## Conclusions

This simple experiment is a humble reminder of the integral relationship between animal and plant life on Earth, in which the former owe their existence to the latter. Without the presence of plants within the sealed environment, the concentration of O_2_ would have fallen and CO_2_ concentration would have risen to a point at which human life could no longer be supported. Whilst O_2_ sustains human life and plants maintain its level within the atmosphere with remarkable efficiency, the fundamental role of photosynthesis is arguably taken for granted. Deprived of plants, the subject within the container would have succumbed to the effects of severe hypoxaemia. The experiment reminds us of our total dependency upon plants, and the ecosystem in which they exist.

## Competing interests

The author declares that they have no competing interests.

## Authors’ contributions

AT conceived the idea, and the experiment was designed by AT, AG and DM. The experiment was conducted by DM, KH, EG, GK, AT and IS. Data were analysed by DM, and the manuscript was written by DM, AG and EG. All authors discussed the results and implications and commented on the manuscript at all stages. All authors read and approved the final manuscript.
